# Comparative analysis of efficacy of different combination therapies of α-receptor blockers and traditional Chinese medicine external therapy in the treatment of chronic prostatitis/chronic pelvic pain syndrome: Bayesian network meta-analysis

**DOI:** 10.1371/journal.pone.0280821

**Published:** 2023-04-20

**Authors:** Kaiyu Zhang, Yi Zhang, Shengwei Hong, Yutian Cao, Chengjiang Liu

**Affiliations:** 1 The First Clinical College, Nanjing University of Chinese Medicine, Nanjing, Jangsu, China; 2 Department of General Medicine, Affiliated Anqing First People’s Hospital of Anhui Medical University, Anqing, Anhui, China; University of Campania Luigi Vanvitelli: Universita degli Studi della Campania Luigi Vanvitelli, ITALY

## Abstract

**Background:**

Combination therapy of α-receptor blockers (α-RBs) and traditional Chinese medicine external therapy can serve as a treatment of chronic prostatitis/chronic pelvic pain syndrome (CP/CPPS). α-RBs includes tamsulosin, terazosin and so on and the traditional Chinese medicine external therapy includes needling, moxibustion, acupoint catgut embedding, acupoint application, auricular point sticking and hot medicated compress and so forth. Currently, there is no study in which Bayesian network meta-analysis is applied to making a comparative analysis of efficacy of different combination therapies of α-RBs and traditional Chinese medicine external therapy in the treatment of CP/CPPS. Therefore, based on Bayesian algorithm, a network meta-analysis was conducted by us to make a comparison between different combination therapies of α-RBs and traditional Chinese medicine external therapy.

**Methods:**

A document retrieval was conducted in the databases PubMed, Cochrane Library, Embase, Web of science, China National Knowledge Infrastructure, WanFang Data Dissertations of China database, VIP China Science and Technology Journal Database, SinoMed. Literatures were searched for published in biomedical journals concerning clinical study on α-RBs combined with various traditional Chinese medicine external therapies in the treatment of CP/CPPS from inception of database to July 2022. Newest version risks of bias assessment tool (RoB2) was used to assess the risks of bias of studies included in this analysis. Stata 16.0 software and R4.1.3 software were used to make a Bayesian network meta-analysis and charts.

**Results:**

19 literatures were included involving 1739 patients concerning 12 interventions which were used in the treatment of CP/CPPS. With respect to the total effective rate, α-RBs+ needling was most likely to be the optimal treatment. Concerning National Institutes of Health Chronic Prostatitis Symptom Index (NIH-CPSI) total score, α-RBs+ moxibustion+ auricular point sticking was most likely to be optimal treatment, the therapy ranking second was α-RBs+ needling, and the therapy ranking third was α-RBs+ moxibustion. Pain score, voiding score and quality-of-life score are subdomains of the NIH-CPSI total score. With regard to pain score, α-RBs+ moxibustion was most likely to be optimal treatment. In reference to voiding and quality-of-life score, there was no statistically significant difference between the efficacy of various interventions.

**Conclusions:**

α-RBs+ needling, α-RBs+ moxibustion and α-RBs+ moxibustion+ auricular point sticking provided relatively good efficacy in the treatment of CP/CPPS. In these treatments, attention should be paid on α-RBs+ needling and α-RBs+ moxibustion which ranked higher many times in the evaluation of various outcome indicators. However, there still were certain limitations in this study, so large-sample clinical randomized control trials with a rigor design following the evidence-based medicine standards need to be conducted to justify the results of this study.

**Systematic review registration:**

[https://www.crd.york.ac.uk/prospero/], identifier: [CRD42022341824].

## Introduction

Prostatitis is one of the most common urologic diseases and is a common cause for physician visit, which National Institutes of Health (NIH) divided into four categories, acute bacterial prostatitis (category I), chronic bacterial prostatitis (category II), chronic prostatitis (CP)/ chronic pelvic pain syndrome (CPPS, category III, inflammatory IIIA, non-inflammatory, IIIB) and asymptomatic inflammatory prostatitis (category IV) [1, 2].

Incidence of CP/CPPS is about 9%~16% in the world, symptoms of which nearly 50% of males experienced [3]. CP/CPPS usually was complicated with negative emotions and lower urinary tract dysfunction and (or) sexual dysfunction [4], which seriously affects the quality of life and mental health state of patients.

α-RBs was widely applied to the treatment of chronic non-bacterial prostatitis nowadays [5]. Although functioning as the main drug of treatment in clinical practice, α-RBs cannot produce significant beneficial effectiveness relative to placebo [6]. In addition, remission of CP/CPPS occurs only during the period of administrating the α-RBs, of which withdrawal tends to lead to extinction of the effect; long-term use enhances the risk of incidence of adverse events [7–9].

Traditional Chinese medicine external therapy, as a common type of non-drug treatment of CP/CPPS, can effectively relieve pelvic pain or discomfort and lower urinary tract symptoms [10–12]. Traditional Chinese medicine external therapy mainly includes needling, moxibustion, acupoint catgut embedding, acupoint application, auricular point sticking and hot medicated compress and so on. Currently, there is no Bayesian network meta-analysis specific to make a comparative analysis of clinical efficacy of different combination therapies of α-RBs and traditional Chinese medicine external therapy in the treatment of CP/CPPS in the world. Based on Bayesian algorithm, a network meta-analysis was conducted in this study to make a comparison and rank between clinical efficacy of different combination therapies of α-RBs and traditional Chinese medicine external therapy in the treatment of CP/CPPS, which was expected to offer evidence-based medicine rationale for clinical application of these combination therapies.

## Methods

This study was reported in accordance with the Preferred Reporting Items for Systematic Reviews and Meta-Analysis (PRISMA) statement and registered with PROSPERO, CRD42022341824 [13].

### Inclusion and exclusion criteria

Inclusion criteria were defined as follows:

1. Subjects diagnosed with CP/CPPS [14, 15];
2. Interventions against the experimental group was α-RBs (for example, tamsulosin and terazosin) combined with traditional Chinese medicine external therapy (for example, needling, moxibustion, acupoint catgut embedding, acupoint application, auricular point sticking and hot medicated compress and so on);
3. Intervention against control group was α-RBs alone or traditional Chinese medicine external therapy alone;
4. Primary outcome indicators included total effective rate and National Institutes of Health Chronic Prostatitis Symptom Index (NIH-CPSI) total score, secondary outcome indicators included NIH-CPSI pain score, NIH-CPSI voiding score and quality-of-life score;
5. Randomized control study.

Exclusion criteria:

1. Studies involving patients complicated with sever psychic disorder and medical condition;
2. Studies with missing outcome data.

### Retrieval strategy

A document retrieval was conducted in the databases PubMed, Cochrane Library, Embase, Web of science, China National Knowledge Infrastructure, WanFang Data Dissertations of China database, VIP China Science and Technology Journal Database, SinoMed. Literatures were searched concerning clinical studies on α-RBs combined with various traditional Chinese medicine external therapies in the treatment of CP/CPPS from inception of database to July 2022. According to criteria provided by Cochrane Collaboration, related document retrieval was conducted by the combination of manual work and using computer [16]. Literature search was conducted according to chronological sequence in reverse order, and there was no language restriction. Search term were searched with a free-text protocol and included: Chronic Prostatitis, Chronic Pelvic Pain Syndrome, Needle, Needling, Acupuncture, Electroacupuncture, Acupuncture and Moxibustion, Warm Needle, Warm Needling, Fire Needle, Needle Knife, Bloodletting, Moxibustion, Hot Medicated Compress, Auricular Point Sticking, Pressing beans on ear points, Auricular points plaster therapy, Acupoint Application, Acupoint Catgut Embedding, Catgut embedding at acupoints, catgut implantation at acupoint, α-antagonists, α receptor antagonist, α adrenergic antagonists, α blocker, Tamsulosin, Flomax, Terazosin, Doxazosin, Naphidil and Selodocin.

### Data extraction and quality assessment

Two researchers (KZ, YZ) screened the literatures and excluded the literatures with insufficient key information, literatures indicating results with missing outcome data and literatures with administration of additional interventions that are inconsistent with the trial protocol by reading the abstract of literatures, the disagreement was arbitrated by a third researcher (SH). After the literature screening, these three researchers will be responsible for data extraction. Data extracted included: study author, year of publication, sample size and intervention, age, prostatitis type, country, duration of treatment, outcome indicators and the definition of "effective". Two researchers assessed respectively the studies which met inclusion and exclusion criteria independently by using newest version risks of bias assessment tool (Version 2 of the Cochrane tool for assessing risk of bias in randomized trial, RoB2) and the disagreement was adjudicated by a third researcher. 2019 revised version RoB2 is structured into 5 domains: bias arising from the randomization process, bias due to deviations from intended interventions, bias due to missing outcome data, bias in measurement of the outcome, bias in selection of the reported result. Responses to each signalling questions are mapped to a proposed judgement which is Yes (Y), Probably Yes (PY), Probably No (PN) and No Information (NI). According to responses of assessors to the signaling questions, one of three levels was assigned to risk of bias for each domain and overall: low risk of bias, some concerns and high risk of bias [17].

### Data analysis

Stata 16.0 software was used to make evidence map of the Bayesian network meta-analysis and funnel plot for comparison-correction of publication bias. R4.1.3 software was used to make Network meta-analysis and Bayesian-Markov Chain Monte Carlo framework was constructed by its getmc and rjags program package. Random-effect mode was fitted with both continuous variable and dichotomous variable, the number of chains of each model all were 4, the number of iterations was 50000 and the number of annealing was 20000. With respect to outcome indicators, odds ratio (OR) was used as effect index of dichotomous variable or count data, mean differences (MD) was used as effect index of continuous variable or measurement data and confidence interval (CI) of each effect index was 95% CIs. Consistency (consistency between direct evidence and indirect evidence) was evaluated statistically in this study and Brooks-Gelman-Rubin methods was used to secure the convergence of each comparison. In addition, consistency model was used to analyze the ranking probability. Ranking probability was calculated with surface under the cumulative ranking curve and average rank. The range of SUCRA was 0~1and the bigger the value was, the higher its predictive value was.

## Results

### Study selection

**Fig 1:**
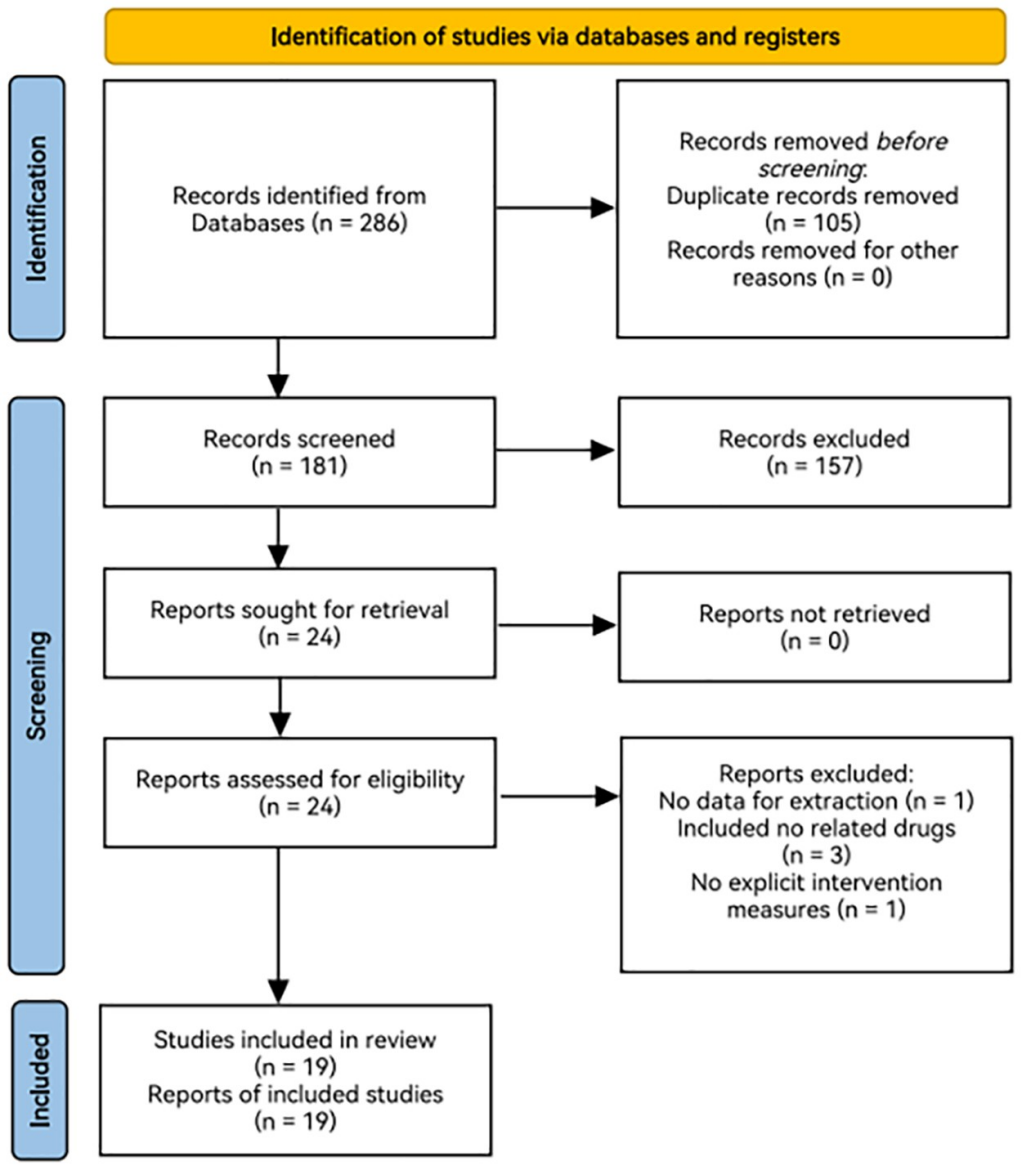
Study flowchart.

There were 181 literatures which were correlated with this study after the repeated literatures were removed in 286 literatures which were attained from databases. According to inclusion and exclusion criteria developed in the previous stage, finally 19 literatures were included meeting the criteria after two researchers screened literatures and excluded the literatures with insufficient key information, literatures indicating results with missing outcome data and literatures with administration of additional interventions that are inconsistent with the trial protocol by reading the abstract of these literatures. Procedures of document retrieval was showed in Fig 1 and characteristics of included studies were seen in Table 1.

**Table 1:** Characteristics of included studies.

Study ID	Sample size and intervention	Age	Prostatitis type	Country	Duration of treatment	Outcome	The definition of "effective"
Hui Li, [18] 2010	30 α-RBs	31.50±10.51	CP/CPPS	China	4 weeks	Clinical effective rate, NIH-CPSI total score.	Symptoms and prostatic fluid examination indicators improved, and the NIH-CPSI total score decreased by ≥ 50%.
	30 Needling	31.40±10.32					
	30 α-RBs+Needling	30.70±9.85					
Guoqin Wang, [19] 2011	25 α-RBs	NR	IIIB	China	30 days	Clinical effective rate, NIH-CPSI total score, Voiding score.	Symptoms improved and the NIH-CPSI total score decreased by ≥ 30%.
	25 Acupoint catgut embedding						
	25 α-RBs+Acupoint catgut embedding						
Xingliang Qi, [20] 2012	30 α-RBs	34.77±10.88	CP/CPPS	China	1 months	NIH-CPSI total score, Pain score, Voiding score, Quality-of-Life score.	NR
	30 α-RBs+Needling	32.60±7.04					
Xinguo Wang, [21] 2013	30 α-RBs	NR	CP/CPPS	China	8 weeks	Clinical effective rate.	Symptoms and prostatic fluid examination indicators improved, and the NIH-CPSI total score decreased by ≥ 30%.
	30 Acupoint application						
	30 α-RBs+Acupoint application						
Yue Wang, [22] 2014	50 α-RBs	36.40±11.40	CP/CPPS	China	4 weeks	Clinical effective rate, NIH-CPSI total score, Pain score, Voiding score, Quality-of-Life score.	The NIH-CPSI total score decreased by ≥ 30%.
	50 α-RBs+Electroacupuncture	35.30±12.40					
Caiping Wang, [23] 2014	35 α-RBs	27.68±3.60	CP/CPPS	China	4 weeks	Clinical effective rate, NIH-CPSI total score.	The NIH-CPSI total score decreased by ≥5 points.
	45 α-RBs+Moxibustion+Auricular point sticking	28.42±4.36					
Shan Gao, [24] 2015	46 α-RBs	66±4	CP/CPPS	China	4 weeks	Clinical effective rate.	The NIH-CPSI total score decreased by ≥ 25%.
	47 α-RBs+Acupuncture	63±6					
Guodong Li, [25] 2015	40 α-RBs	49.20±11.50	CP/CPPS	China	4 weeks	Clinical effective rate, NIH-CPSI total score, Pain score	The NIH-CPSI total score decreased by ≥ 25%.
	40 α-RBs+Moxibustion	50.30±9.70					
Ruimin Zhang, [26] 2017	45 α-RBs	48.02±9.16	CP/CPPS	China	2 weeks	Clinical effective rate, NIH-CPSI total score.	Symptoms and prostatic fluid examination indicators improved.
	45 α-RBs+Acupoint application	47.71±8.13					
Jiang Guo, [27] 2018	120 α-RBs	NR	CP/CPPS	China	30 days	Clinical effective rate, NIH-CPSI total score, Pain score, Voiding score, Quality-of-Life score.	The NIH-CPSI total score decreased by ≥ 25%.
	120 α-RBs+Moxibustion						
Biaotai Li, [28] 2018	50 α-RBs	32.20±3.90	CP/CPPS	China	20 days	Clinical effective rate, NIH-CPSI total score.	Symptoms and prostatic fluid examination indicators improved.
	50 α-RBs+Moxibustion	32.40±3.70					
Shuying Zhang, [29] 2018	60 α-RBs	25.22±6.97	CP/CPPS	China	2 weeks	NIH-CPSI total score, Quality-of-Life score.	The NIH-CPSI total score decreased by ≥ 30%.
	60 α-RBs+Hot medicated compress	24.65±7.59					
Yanxia Zhu, [30] 2019	30 α-RBs	43.00±11.90	CP/CPPS	China	8 weeks	Clinical effective rate, NIH-CPSI total score.	Symptoms and prostatic fluid examination indicators improved.
	30 α-RBs+Needling	42.60±11.80					
Yuanzhu Chen, [31] 2019	18 α-RBs	38.25±4.63	CP/CPPS	China	3 weeks	Clinical effective rate, NIH-CPSI total score, Pain score, Voiding score, Quality-of-Life score.	Symptoms improved and the NIH-CPSI total score decreased by ≥ 30%.
	18 α-RBs+Needling	36.81±4.35					
Wei Gao, [32] 2020	50 α-RBs	32.30±9.20	CP/CPPS	China	12 weeks	Clinical effective rate, NIH-CPSI Total score, Pain score, Voiding score, Quality-of-Life score.	The NIH-CPSI total score decreased by ≥ 30%.
	50 α-RBs+Moxibustion	30.20±8.90					
Li Zeng, [33] 2021	15 α-RBs	54.53±5.62	CP/CPPS	China	2 weeks	Clinical effective rate, NIH-CPSI total score.	Symptoms improved and the NIH-CPSI total score decreased by ≥ 30%.
	15 α-RBs+Moxibustion	52.80±7.46					
Qifang Liang, [34] 2021	30 α-RBs	42±13	CP/CPPS	China	90 days	Clinical effective rate, NIH-CPSI total score.	Symptoms have improved.
	31 α-RBs+Electroacupuncture	42±13					
Zhiping Wang, [35] 2021	42 α-RBs	41.93±4.13	CP/CPPS	China	4 weeks	Clinical effective rate.	Symptoms and prostatic fluid examination indicators improved.
	42 α-RBs+Acupoint application	42.11±4.32					
Jungang Lu, [36] 2021	75 α-RBs	33.60±1.98	IIIB	China	4 weeks	Clinical effective rate, NIH-CPSI total score, Pain score, Voiding score, Quality-of-Life score.	Symptoms have improved.
	75 α-RBs+Acupoint catgut embedding	36.04±0.27					

α-RBs, α-Receptor Blockers; CP/CPPS, Chronic prostatitis/Chronic pelvic pain syndrome; NIH-CPSI, National Institutes of Health Chronic Prostatitis Symptom Index.

### Study description

**Fig 2:**
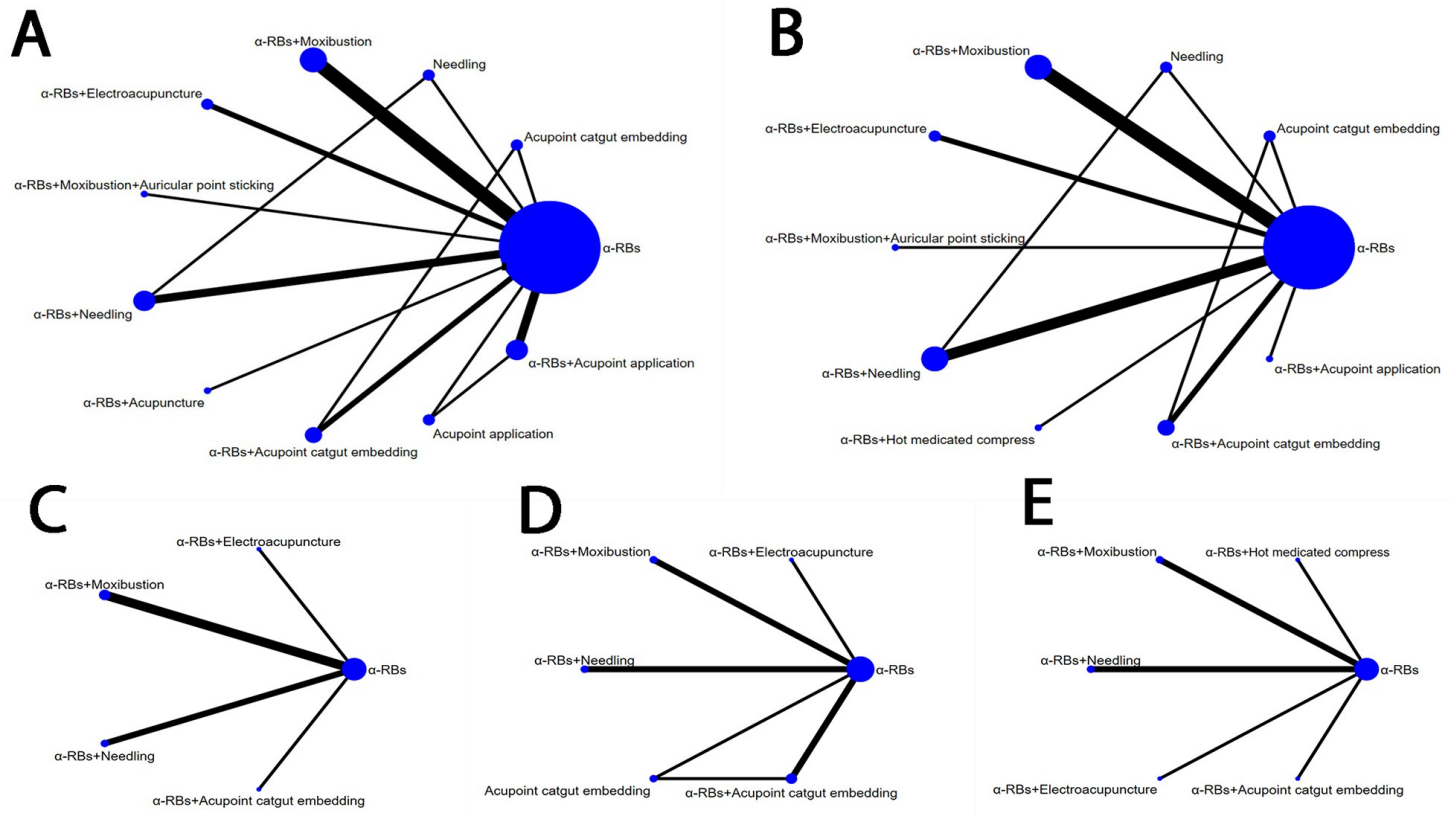
Network graphs for various outcomes. (A) Clinical effective rate;(B) NIH-CPSI Total Score;(C) Pain Score;(D) Voiding Score;(E) Quality-of-Life Score. α-RBs, α-Receptor Blockers.

19 literatures [18–36] were included involving 1739 patients (833 cases in experimental group and 906 in control group) concerning 12 interventions used in the treatment of CP/CPPS. Range of age was about 20–70 years old and most were young and mid aged patients. In these studies, α-RBs + moxibustion vs α-RBs (n = 5), α-RBs+ electroacupuncture vs α-RBs (n = 2), α-RBs +needling vs α-RBs vs needling (n = 1), α-RBs + needling vs α-RBs (n = 3), α-RBs +acupuncture vs α-RBs (n = 1), α-RBs + moxibustion+ auricular point sticking vs α-RBs (n = 1), α-RBs + hot medicated compress vs α-RBs (n = 1), α-RBs + acupoint catgut embedding vs α-RBs (n = 1), α-RBs + acupoint catgut embedding vs α-RBs vs acupoint catgut embedding (n = 1), α-RBs + acupoint application vs α-RBs (n = 2), α-RBs + acupoint application vs α-RBs vs acupoint application (n = 1). All of the studies were from China and the duration of treatment was 2 weeks to 3 months, while most of the studies lasted 4 weeks. Network graphs of various outcome indicators were seen in Fig 2.

### Quality assessment

**Fig 3:**
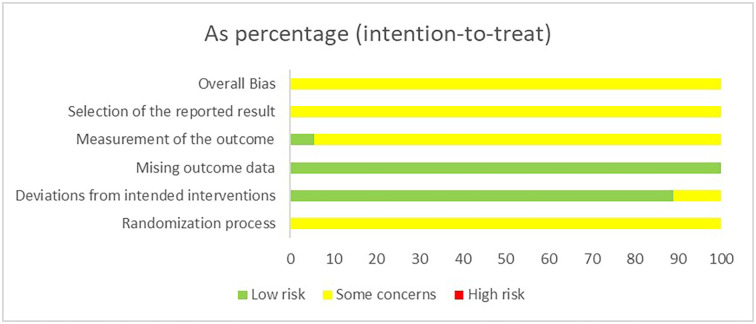
Risk of bias graph.

Two researchers assessed respectively the risk of bias of the studies included by using newest version risks of bias assessment tool (Version 2 of the Cochrane tool for assessing risk of bias in randomized trial, RoB2). Assessing items were as follows:

1. There existed some concerns arising from the randomization process for all studies [18–36], 11 studies [18, 19, 21, 22, 25, 27, 29, 30, 32, 33, 35] did not disclose specific randomization methods, 10 studies [18–22, 25–27, 29, 32] did not disclose the allocation hiding or not, and 9 studies [23, 24, 28, 30, 31, 33–36] did not carry out allocation hiding;
2. There were low risk due to deviations from intended interventions for 17 studies and some concerns for 2 studies [24, 34] (dropout);
3. All studies were at low risk due to missing outcome data (data for outcomes were available for all participants randomized);
4. One study had low risk in measurement of the outcome and 18 studies had some concerns in measurement of the outcome (outcome assessors were not blinded) [34];
5. There existed some concerns in selection of the reported result for all studies (pre-specified plan that was finalized before unblinded outcome data were available for analysis was unclear). Risk of bias graphs were seen in Fig 3.

### Outcomes

#### Clinical effective rate

17 studies were included in total (n = 1559) involving 11 interventions. At the end of course of therapy, α-RBs combined with traditional Chinese medicine external therapy was superior to α-RBs alone or traditional Chinese medicine external therapy alone with respect to the indicators of clinical effective rate. Based on results of network meta-analysis, α-RB+ needling (OR: -2.32;95%CrI, -3.52 to -1.31) was superior to α-RBs alone. There was statistical significance in the difference between the above interventions (P<0.05) while there was no statistical significance in the difference between the other interventions. According to the ranking possibility, α-RBs+ needling was ranked No. 1 (95.10%). Results of comparison and rank between interventions were shown in Table 2.

**Table 2:** Results of network meta-analysis. (Clinical effective rate).

Clinical effective rate										
**Needling (SUCRA = 42.49%)**										
0.92 (-0.88, 2.77)	**Acupoint catgut embedding (SUCRA = 13.89%)**									
0.94 (-0.73, 2.56)	0.02 (-1.81, 1.77)	**Acupoint application (SUCRA = 12.55%)**								
0.75 (-0.42, 1.94)	-0.18 (-1.54, 1.18)	-0.19 (-1.34, 1.01)	**α-RBs (SUCRA = 14.93%)**							
-0.68 (-1.98, 0.66)	-1.59 (-3.09, -0.13)	-1.61 (-2.90, -0.27)	-1.42 (-2.02, -0.84)	**α-RBs+Moxibustion (SUCRA = 70.48%)**						
-0.47 (-2.32, 1.32)	-1.39 (-3.43, 0.52)	-1.42 (-3.22, 0.38)	-1.21 (-2.65, 0.14)	0.21 (-1.35, 1.70)	**α-RBs+Moxibustion +Auricular point sticking (SUCRA = 60.57%)**					
-0.23 (-1.67, 1.24)	-1.14 (-2.76, 0.42)	-1.15 (-2.58, 0.33)	-0.97 (-1.86, -0.09)	0.45 (-0.61, 1.52)	0.24 (-1.38, 1.95)	**α-RBs+ Electroacupuncture (SUCRA = 50.75%)**				
-0.72 (-2.35, 0.84)	-1.65 (-3.23, -0.15)	-1.67 (-3.27, -0.07)	-1.46 (-2.63, -0.44)	-0.04 (-1.34, 1.14)	-0.26 (-2.03, 1.49)	-0.50 (-1.95, 0.85)	**α-RBs+Acupoint catgut embedding (SUCRA = 71.10%)**			
-0.67 (-2.11, 0.78)	-1.59 (-3.20, -0.01)	-1.60 (-2.87, -0.35)	-1.41 (-2.30, -0.58)	0.01 (-1.08, 1.04)	-0.20 (-1.84, 1.47)	-0.44 (-1.69, 0.77)	0.06 (-1.32, 1.52)	**α-RBs+Acupoint application (SUCRA = 69.44%)**		
-1.58 (-2.96, -0.30)	-2.50 (-4.28, -0.81)	-2.51 (-4.16, -0.94)	**-2.32 (-3.52, -1.31)**	-0.90 (-2.23, 0.28)	-1.11 (-2.90, 0.68)	-1.35 (-2.83, -0.01)	-0.86 (-2.40, 0.72)	-0.90 (-2.38, 0.45)	**α-RBs+Needling (SUCRA = 95.10%)**	
-0.15 (-2.01, 1.61)	-1.07 (-3.08, 0.86)	-1.08 (-2.92, 0.69)	-0.89 (-2.31, 0.43)	0.53 (-1.00, 1.99)	0.32 (-1.60, 2.29)	0.07 (-1.57, 1.65)	0.58 (-1.20, 2.33)	0.52 (-1.14, 2.18)	1.42 (-0.28, 3.23)	**α-RBs+Acupuncture (SUCRA = 48.70%)**

α-RBs, α-Receptor Blockers

#### NIH-CPSI total score

16 studies were included in total (n = 1472) involving 10 interventions. At the end of course of therapy, in reference to indicators of NIH-CPSI total score, α-RBs combined with traditional Chinese medicine external therapy was superior to α-RBs alone or traditional Chinese medicine external therapy alone. Based on results of network meta-analysis, α-RBs+ moxibustion (MD:5.04;95%CrI,2.19 to 7.71), α-RBs+ moxibustion+ auricular point sticking (MD:6.59;95%CrI,0.66 to 12.56), α-RBs+ electroacupuncture (MD:4.80;95%CrI,0.35 to 9.56) and α-RBs+ needling (MD:5.56;95%CrI,2.21 to 8.93) were all superior to α-RBs alone. There were statistical significance in difference between the above interventions (P<0.05) while there were no statistical significance in difference between the other interventions. According to the ranking possibility, α-RBs+ moxibustion+ auricular point pressing (79.21%) was ranked No. 1 and the therapy ranking second was α-RBs+ needling (72.54%) and the therapy ranking third was α-RBs+ moxibustion (65.93%). Results of comparison and rank between interventions were shown in Table 3.

**Table 3:** Results of network meta-analysis. (NIH-CPSI Total Score).

NIH-CPSI Total Score									
**Needling (SUCRA = 45.63%)**									
-2.42 (-10.86, 5.97)	**Acupoint catgut embedding (SUCRA = 21.77%)**								
-3.36 (-9.45, 2.68)	-0.94 (-6.76, 4.87)	**α-RBs (SUCRA = 9.04%)**							
1.67 (-5.07, 8.25)	4.09 (-2.44, 10.47)	**5.04 (2.19, 7.71)**	**α-RBs+Moxibustion (SUCRA = 65.93%)**						
3.23 (-5.28, 11.76)	5.66 (-2.68, 13.95)	**6.59 (0.66, 12.56)**	1.54 (-4.91, 8.23)	**α-RBs+Moxibustion +Auricular point sticking (SUCRA = 79.21%)**					
1.45 (-6.06, 9.20)	3.85 (-3.45, 11.46)	**4.80 (0.35, 9.56)**	-0.24 (-5.36, 5.38)	-1.79 (-9.20, 5.88)	**α-RBs+ Electroacupuncture (SUCRA = 61.17%)**				
0.01 (-8.64, 8.54)	2.40 (-6.04, 10.87)	3.36 (-2.75, 9.44)	-1.69 (-8.28, 5.10)	-3.24 (-11.81, 5.24)	-1.45 (-9.26, 6.05)	**α-RBs+Hot medicated compress (SUCRA = 45.74%)**			
0.92 (-6.53, 8.35)	3.34 (-2.52, 9.17)	4.29 (-0.03, 8.61)	-0.76 (-5.78, 4.50)	-2.31 (-9.69, 5.02)	-0.52 (-7.02, 5.65)	0.93 (-6.55, 8.35)	**α-RBs+Acupoint catgut embedding (SUCRA = 56.27%)**		
-0.40 (-9.08, 8.20)	2.01 (-6.50, 10.54)	2.97 (-3.22, 9.16)	-2.08 (-8.75, 4.79)	-3.64 (-12.23, 4.97)	-1.83 (-9.72, 5.75)	-0.40 (-9.08, 8.30)	-1.32 (-8.87, 6.19)	**α-RBs+Acupoint application (SUCRA = 41.69%)**	
2.21 (-3.92, 8.30)	4.61 (-2.13, 11.33)	**5.56 (2.21, 8.93)**	0.52 (-3.71, 4.99)	-1.03 (-7.90, 5.82)	0.75 (-5.13, 6.33)	2.20 (-4.73, 9.19)	1.28 (-4.17, 6.76)	2.60 (-4.47, 9.66)	**α-RBs+Needling (SUCRA = 72.54%)**

α-RBs, α-Receptor Blockers; NIH-CPSI, National Institutes of Health Chronic Prostatitis Symptom Index.

#### Pain score

7 studies were included in total (n = 766) including 5 interventions. At the end of course of therapy, in reference to indicators of NIH-CPSI pain score, α-RBs combined with traditional Chinese medicine external therapy was superior to α-RBs alone or traditional Chinese medicine external therapy alone. α-RBs+ moxibustion (MD:2.86;95%CrI,0.21 to 5.57) was superior to α-RBs alone. There was statistical significance in the difference between the above interventions (P<0.05) while there was no statistical significance in the difference between the other interventions. According to ranking probability, α-RBs+ moxibustion (77.93%) was ranked No.1. Results of comparison and rank between interventions were shown in Table 4.

**Table 4:** Results of network meta-analysis. (Pain Score).

Pain Score				
**α-RBs (SUCRA = 16.81%)**				
**2.86 (0.21, 5.57)**	**α-RBs+Moxibustion (SUCRA = 77.93%)**			
1.59 (-3.00, 6.20)	- 1.27 (-6.58, 4.05)	**α-RBs+ Electroacupuncture (SUCRA = 51.85%)**		
0.45 (-4.07, 4.96)	-2.41 (-7.69, 2.84)	- 1. 14 (-7.63, 5.26)	**α-RBs+Acupoint catgut embedding (SUCRA = 31.35%)**	
2.62 (-0.72, 5.78)	-0.25 (-4.53, 3.88)	1.03 (-4.71, 6.56)	2.17 (-3.47, 7.65)	**α-RBs+Needling (SUCRA = 72.06%)**

α-RBs, α-Receptor Blockers

#### Voiding score

7 studies (n = 761) were included in total involving 6 interventions. At the end of course of therapy, in reference to indicators of NIH-CPSI voiding score, α-RBs combined with traditional Chinese medicine external therapy was superior to α-RBs alone. However, there was no statistical significance in the difference between various interventions (P>0.05). Results of comparison and rank between interventions were shown in Table 5.

**Table 5:** Results of network meta-analysis. (Voiding Score).

Voiding Score					
**Acupoint catgut embedding (SUCRA = 23.34%)**					
0.05 (-3.78, 4.00)	**α-RBs (SUCRA = 18 39%)**				
2.76 (- 1.99, 7.57)	2.70 (-0. 13, 5.48)	**α-RBs+Moxibustion (SUCRA = 81.14%)**			
1.56 (-3.92, 7.20)	1.51 (-2.45, 5.51)	- 1.20 (-5.99, 3.72)	**α-RBs+ Electroacupuncture (SUCRA = 55.21%)**		
2.29 (- 1.57, 6. 11)	2.24 (-0.72, 5.07)	-0.47 (-4.53, 3.53)	0.73 (-4.26, 5.56)	**α-RBs+Acupoint catgut embedding (SUCRA = 72.43%)**	
1.27 (-3.49, 6. 16)	1.22 (- 1.63, 4.06)	- 1.48 (-5.45, 2.55)	-0.29 (-5.21, 4.56)	- 1.01 (-5.03, 3. 11)	**α-RBs+Needling (SUCRA = 49.48%)**

α-RBs, α-Receptor Blockers

#### Quality-of-Life score

7 studies (n = 806) were included in total including 6 interventions. At the end of course of therapy, in reference to indicators of NIH-CPSI quality-of-life score, α-RBs combined with traditional Chinese medicine external therapy was superior to α-RBs alone. However, there was no statistical significance in the difference between various interventions (P>0.05). Results of comparison and rank between interventions were shown in Table 6.

**Table 6:** Results of network meta-analysis. (Quality-of-Life Score).

Quality-of-Life Score					
**α-RBs (SUCRA = 13.58%)**					
1.55 (-1.05, 4.10)	**α-RBs+Moxibustion (SUCRA = 55.68%)**				
0.51 (-3.17, 4.14)	-1.05 (-5.49, 3.45)	**α-RBs+ Electroacupuncture (SUCRA = 30.65%)**			
1.89 (-1.79, 5.55)	0.34 (-4.14, 4.82)	1.39 (-3.81, 6.56)	**α-RBs+Hot medicated compress (SUCRA = 63.46%)**		
1.65 (-1.96, 5.27)	0.09 (-4.31, 4.58)	1.15 (-3.99, 6.28)	-0.25 (-5.38, 4.92)	**α-RBs+Acupoint catgut embedding (SUCRA = 57.53%)**	
2.49 (-0.20, 5.03)	0.95 (-2.78, 4.55)	1.99 (-2.58, 6.44)	0.61 (-3.95, 5.04)	0.85 (-3.66, 5.25)	**α-RBs+Needling (SUCRA = 79.10%)**

α-RBs, α-Receptor Blockers

#### Safety

In 19 studies, adverse reaction was not mentioned in 14 studies, the existence of adverse reaction was denied in one study and the incidence of adverse reaction in the most of experimental group was less than that of control group in the remaining 4 studies, which suggested that safety of combination therapy of α-RBs and traditional Chinese medicine external therapy was relatively good.

#### Publication bias

**Fig 4:**
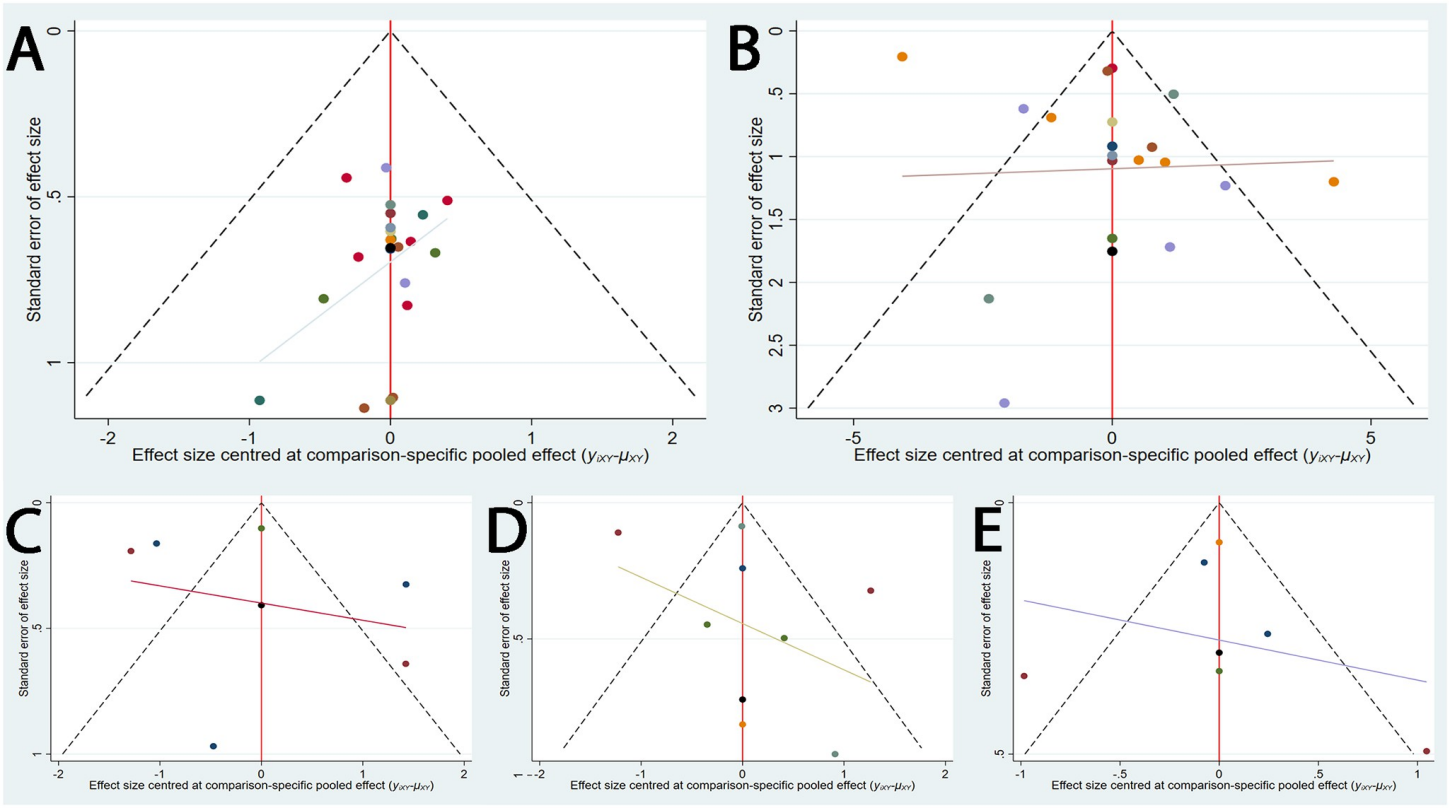
Funnel plots (A) Clinical effective rate;(B) NIH-CPSI Total Score;(C) Pain Score;(D) Voiding Score;(E) Quality-of-Life Score.

Funnel plots of 5 results were shown in Fig 4 which were used to evaluate publication bias. There may exist certain publication bias, which was suggested by symmetry of all funnel plots which was not good enough.

## Discussion

Bayesian algorithm has certain advantages over frequentists’: confidence interval got by frequentists’ algorithm cannot be explained by possibility and framing of decision supporting the decision is incorporated into the Bayesian algorithm [37]. When the numbers of studies are modicum, the credibility of results achieved by Bayesian algorithm is higher than that by frequentists’ algorithm.

Effect of 12 common interventions against CP/CPPS were evaluated systematically by Bayesian network meta-analysis with 19 related studies and 5 outcome indicators in this study. 12 interventions can be divided into three types in general (α-RBs alone, traditional Chinese medicine external therapy alone and combination therapy).

Based on the results of network meta-analysis, there was no significant difference between α-RBs alone and traditional Chinese medicine external therapy alone while combination therapy of α-RBs and traditional Chinese medicine external therapy was superior to α-RBs alone or traditional Chinese medicine external therapy alone with respective to all outcomes. With regard to clinical effective rate, the efficacy of α-RBs+ needling was significantly superior to that of α-RBs. As for NIH-CPSI total score, the efficacy of α-RBs+ moxibustion, α-RBs+ moxibustion+ auricular point sticking, α-RBs+ electroacupuncture, and α-RBs+ needling were significantly superior to that of α-RBs. In regard to pain score, the efficacy of α-RBs+ moxibustion was significantly superior to that of α-RBs. The above difference had statistical significance (P<0.05). With respect to voiding and quality-of-life score, there was no significant difference between the efficacy of combination therapies of α-RBs and traditional Chinese medicine external therapy and the use of α-RBs alone or traditional Chinese medicine external therapy alone (P>0.05).

In reference to total effective rate, α-RBs+ needling was most likely to be optimal treatment. Concerning NIH-CPSI total score, α-RBs+ moxibustion+ auricular point sticking was most likely to be optimal treatment, the therapy ranking second was α-RBs+ needling, and the therapy ranking third was α-RBs+ moxibustion. In regard to pain score, α-RBs+ moxibustion was most likely to be optimal treatment. In reference to voiding and quality-of-life score, there was no statistically significant difference between the efficacy of various interventions. In summary, α-RBs+ needling, α-RBs+ moxibustion, and α-RBs+ moxibustion+ auricular point sticking, provided relatively good clinical efficacy. In these treatments, attention should be paid on α-RBs+ needling and α-RBs+ moxibustion which ranked higher many times in the evaluation of various outcome indicators.

CP/CPPS falls into category III prostatitis of National Institutes of Health (NIH) classification, of which the definition is that pelvic and urinary pain or discomfort lasts for at least 3 months within first 6 months which is associated with urinary symptoms and no proof of bacterial infection [4]. The pathogenesis of CP/CPPS may include infection, reflux, defective function of urothelial of lower urinary tract, anxiety depressive state, pelvis-related disease, inflammatory, immunology response, etc. This is a question worth discussing due to perplexing etiology [38–43]. In current clinical practice, there remain to be no gold standard of diagnostic tests, so the diagnosis of CP/CPPS is commonly based on exclusive diagnosis methods [2, 44, 45]. Therefore, effectiveness of treatment of CP/CPPS is also unsatisfactory [46].

α-RBs is an important adjuvant tool in the clinical treatment of CP/CPPS [47]. Bladder neck and prostate gland are rich in α receptors which are in the central nervous system and are related to chronic pain syndrome. α-RBs block the α adrenergic receptors in the prostate selectively to make the smooth muscle of prostate relax resulting in promoting voiding of urinary bladder and improving the symptom of dysuria as well as interact with sympathetic nerve of pelvic diaphragm to alleviate tension pain and reduce the incidence of lower urinary tract neurogenic inflammation [47–49]. Needling and moxibustion are traditional types of therapy of traditional Chinese medicine external therapies. In 1997, the value of needling in the treatment of pain and/or inflammation resulting from various diseases was recognized by National Institutes of Health [50]. Mechanism of therapeutic effect of needling on CP/CPPS includes the follows: 1. Immunomodulatory effect. Immune-related factors play a crucial role in the pathogenesis of CP/CPPS. Needling can modulate inflammatory factors and immunocytes by downgrading the level of proinflammatory factors, upgrading anti-inflammatory and adjusting inflammatory regulators. Multiple studies showed that needling can activate vagus nerve to inhibit the macrophage activation and production of proinflammatory factors and promote dopamine generation of adrenal medulla, which can control the inflammation [50, 51]. In addition, needling can also lead to an increase in the level of natural killer (NK) cells in the blood which can yield T cell helper factors related to remission of diseases and modulate immune to keep the inflammation in check [52–54]. 2. Improving urodynamics. Voiding dysfunction is one of main symptoms of CP in which pelvic floor muscle dysfunction may play an important role; and spastic pelvic floor syndrome is closely associated with pelvic tenderness of patients with CP/CPPS [55]. Many studies indicated that urodynamics and voiding dysfunction can be improved by acupuncture which regulates pelvic floor muscle contractility [56, 57]. 3. Regulating blood circulation. Most of patients with CP/CPPS suffer from pelvic congestion syndrome with blood circulation and microcirculation dysfunction [58]. Studies showed that needling can improve venous circulation which is suggested by pelvic magnetic resonance venography imaging [59]. Moxibustion features high safety and low cost and so on including moxibustion on governor vessel acupoints, cotton moxibustion, medicinal thread moxibustion and pyretic moxibustion and the such [60]. Studies concerning moxibustion in the treatment of chronic CP/CPPS are relative less nowadays which indicated that the primary mechanism of moxibustion in the treatment of CP/CPPS is to improve whole blood reduced viscosity, white blood cell rigidity index, aggregation index and the level of fibrinogen to ameliorate hemorheology state by exerting thermal stimulation on blood vessel and tissues [28, 61]. When moxibustion is combined with antipyretics-analgesic and anti-inflammatory drugs, anti-inflammatory and analgesic effect can be enhanced because the combination therapy can downgrade inflammatory factors, which is suggested by many studies [62, 63]. Auricular point sticking can significantly and effectively relieve pain, mechanism of which is to increase the concentration of plasma β-endorphin [64, 65]. The above studies provided the rationale about the feasibility of the combination therapy of α-RBs and traditional Chinese medicine external therapy represented by needling, moxibustion, and auricular point sticking in the treatment of CP/CPPS. In previous published papers, it was found that α-RBs alone or traditional Chinese medicine external therapy alone could effectively relieve CP/CPPS symptoms compared with placebo [5, 66, 67]. This study innovatively pointed out that combination therapy of α-RBs and traditional Chinese medicine external therapy was superior to α-RBs alone or traditional Chinese medicine external therapy alone with respective to all outcomes, which can be used for reference by clinicians.

Dealing with CP/CPPS is a challenging task, and we cannot effectively manage CP/CPPS at present. One study showed that placebo can significantly improve CP/CPPS symptoms, and it is believed that anticipatory pain relief is one of the main mechanisms of placebo analgesia, and a series of complementary and alternative therapies such as external treatment of traditional Chinese medicine also have placebo effect to some extent. This suggests that the placebo effect is significant in alleviating CP/CPPS and deserves further study [68, 69].

However, there exist numerous limitations in this study:

First, the heterogeneity of protocols used for each Chinese medicine external therapy limits comparisons between studies;

Second, definition of clinical efficacy varied among the studies limits the comparison; Third, CP/CPPS was composed of two types (inflammatory IIIA, non-inflammatory, IIIB), most of the studies did not specify the type of CP/CPPS, which limited the comparison between studies;

Fourth, most of the results were deduced from indirect comparison;

Fifth, the quality of involving studies may be not high and the number of studies included in the analysis with respected to part of outcome indicators was less, potentially compromising the robustness of the results.

## Conclusion

Based on Bayesian algorithm, network meta-analysis was made to compare the efficacy of 12 interventions in this study. The results of this study implicated: α-RBs+ needling, α-RBs+ moxibustion and α-RBs+ moxibustion+ auricular point sticking had better clinical effect in the treatment of CP/CPPS. In these treatments, attention should be paid on α-RBs+ needling and α-RBs+ moxibustion which ranked higher many times in various outcome indicators. Results of this study can guide and promote the clinical application of α-RBs+ traditional Chinese medicine external therapy. Meanwhile, large-sample clinical randomized control trials with a rigor design following the evidence-based medicine standards need to be conducted to explore the clinical effect of α-RBs+ traditional Chinese medicine external therapy in the treatment of CP/CPPS to achieve a more objective and justified conclusion.

## Supporting information

S1 File Search strategy. (DOCX)

S1 Table PRISMA 2020 checklist. (DOCX)

S2 Table Outcomes as reported in trials included in the network meta-analysis. (XLSX)
