# Supplementary material for: Comparative analysis of efficacy of different combination therapies of α-receptor blockers and traditional Chinese medicine external therapy in the treatment of chronic prostatitis/chronic pelvic pain syndrome: Bayesian network meta-analysis
Source: PLoS One. 2023 Apr 20;18(4):e0280821. doi: 10.1371/journal.pone.0280821 (PMC10118174; doi:10.1371/journal.pone.0280821)
Supplement: S1 File — (DOCX) [file pone.0280821.s001.docx]

**WanFang Data Dissertations of China database（万方）:**全部:(慢性前列腺炎 or 慢性盆腔疼痛综合征) and 全部:(针 or 针刺 or 电针 or 针灸 or 温针 or 火针 or 针刀 or 放血 or 灸 or 熨法 or 耳穴压豆 or 穴位贴敷 or 穴位埋线) and 全部:(α拮抗剂 or α阻滞剂 or α受体拮抗剂 or α肾上腺素受体拮抗剂 or 坦索罗辛 or 坦洛新 or 特拉唑嗪 or 多沙唑嗪 or 萘哌地尔 or 赛洛多辛)

**China National Knowledge Infrastructure（知网）:**主题:(慢性前列腺炎 + 慢性盆腔疼痛综合征) and 主题:(针 + 针刺 + 电针 + 针灸 + 温针 + 火针 + 针刀 + 放血 + 灸 + 熨法 + 耳穴压豆 + 穴位贴敷 + 穴位埋线) and 主题:(α拮抗剂 + α阻滞剂 + α受体拮抗剂 + α肾上腺素受体拮抗剂 + 坦索罗辛 + 坦洛新 + 特拉唑嗪 + 多沙唑嗪 + 萘哌地尔 + 赛洛多辛)

**VIP China Science and Technology Journal Database（维普）:**任意字段:(慢性前列腺炎 or 慢性盆腔疼痛综合征) and 任意字段:(针 or 针刺 or 电针 or 针灸 or 温针 or 火针 or 针刀 or 放血 or 灸 or 熨法 or 耳穴压豆 or 穴位贴敷 or 穴位埋线) and 任意字段:(α拮抗剂 or α阻滞剂 or α受体拮抗剂 or α肾上腺素受体拮抗剂 or 坦索罗辛 or 坦洛新 or 特拉唑嗪 or 多沙唑嗪 or 萘哌地尔 or 赛洛多辛)

**SinoMed（中国生物医学文献服务系统）:**全部字段:(慢性前列腺炎 OR 慢性盆腔疼痛综合征) and 全部字段:(针 OR 针刺 OR 电针 OR 针灸 OR 温针 OR 火针 OR 针刀 OR 放血 OR 灸 OR 熨法 OR 耳穴压豆 OR 穴位贴敷 OR 穴位埋线) and 全部字段:(α拮抗剂 OR α阻滞剂 OR α受体拮抗剂 OR α肾上腺素受体拮抗剂 OR 坦索罗辛 OR 坦洛新 OR 特拉唑嗪 OR 多沙唑嗪 OR 萘哌地尔 OR 赛洛多辛)

**Phumed:**All Fields:(Chronic Prostatitis) OR (Chronic Pelvic Pain Syndrome) and All Fields: (Needle) OR (Needling) OR (Acupuncture) OR (Electroacupuncture) OR (Warm Needle) OR (Warm Needling) OR (Fire Needle) OR (Needle Knife) OR (Bloodletting) OR (Moxibustion) OR (Hot Medicated Compress) OR (Auricular Point Sticking) OR (Pressing beans on ear points) OR (Auricular points plaster therapy) OR (Acupoint Application) OR (Acupoint Catgut Embedding) OR (Catgut embedding at acupoints) OR (catgut implantation at acupoint) and All Fields:(α-antagonists) OR (α receptor antagonist) OR (α adrenergic antagonists) OR (α blocker) OR (Tamsulosin) OR (Flomax) OR (Terazosin) OR (Doxazosin) OR (Naphidil) OR (Selodocin)

**Web of Science:**TS=(((Chronic Prostatitis) OR (Chronic Pelvic Pain Syndrome)) and ((Needle) OR (Needling) OR (Acupuncture) OR (Electroacupuncture) OR (Warm Needle) OR (Warm Needling) OR (Fire Needle) OR (Needle Knife) OR (Bloodletting) OR (Moxibustion) OR (Hot Medicated Compress) OR (Auricular Point Sticking) OR (Pressing beans on ear points) OR (Auricular points plaster therapy) OR (Acupoint Application) OR (Acupoint Catgut Embedding) OR (Catgut embedding at acupoints) OR (catgut implantation at acupoint)) and ((α-antagonists) OR (α receptor antagonist) OR (α adrenergic antagonists) OR (α blocker) OR (Tamsulosin) OR (Flomax) OR (Terazosin) OR (Doxazosin) OR (Naphidil) OR (Selodocin)))

**Embase:**Broad search:((Chronic Prostatitis) OR (Chronic Pelvic Pain Syndrome)) and ((Needle) OR (Needling) OR (Acupuncture) OR (Electroacupuncture) OR (Warm Needle) OR (Warm Needling) OR (Fire Needle) OR (Needle Knife) OR (Bloodletting) OR (Moxibustion) OR (Hot Medicated Compress) OR (Auricular Point Sticking) OR (Pressing beans on ear points) OR (Auricular points plaster therapy) OR (Acupoint Application) OR (Acupoint Catgut Embedding) OR (Catgut embedding at acupoints) OR (catgut implantation at acupoint)) and ((α-antagonists) OR (α receptor antagonist) OR (α adrenergic antagonists) OR (α blocker) OR (Tamsulosin) OR (Flomax) OR (Terazosin) OR (Doxazosin) OR (Naphidil) OR (Selodocin))

**Cochrane Library:**Title Abstract Keyword: ((Chronic Prostatitis) OR (Chronic Pelvic Pain Syndrome)) and ((Needle) OR (Needling) OR (Acupuncture) OR (Electroacupuncture) OR (Warm Needle) OR (Warm Needling) OR (Fire Needle) OR (Needle Knife) OR (Bloodletting) OR (Moxibustion) OR (Hot Medicated Compress) OR (Auricular Point Sticking) OR (Pressing beans on ear points) OR (Auricular points plaster therapy) OR (Acupoint Application) OR (Acupoint Catgut Embedding) OR (Catgut embedding at acupoints) OR (catgut implantation at acupoint)) and ((α-antagonists) OR (α receptor antagonist) OR (α adrenergic antagonists) OR (α blocker) OR (Tamsulosin) OR (Flomax) OR (Terazosin) OR (Doxazosin) OR (Naphidil) OR (Selodocin))
